# Renal Function in Patients with Hypertension Associated Congestive Cardiac Failure Seen in a Tertiary Hospital

**DOI:** 10.1155/2012/769103

**Published:** 2012-10-10

**Authors:** C. U. Osuji, C. U. Nwaneli, B. J. Onwubere, E. I. Onwubuya, G. I. Ahaneku

**Affiliations:** ^1^Department of Medicine, Nnamdi Azikiwe University Teaching Hospital, PMB 5025, Anambra State, Nnewi 435101, Nigeria; ^2^Department of Medicine, University of Nigeria Teaching Hospital, PMB 01129, Enugu State, Enugu 400001, Nigeria

## Abstract

*Background*. Chronic kidney disease is frequently seen in patients with congestive cardiac failure and is an independent risk factor for morbidity and mortality. The aim of this study was to determine the prevalence of chronic kidney disease in patients with hypertension associated congestive cardiac failure. *Method*. One hundred and fifty patients with hypertension associated congestive cardiac failure were recruited consecutively from the medical outpatient department and the medical wards of the Nnamdi Azikiwe University Teaching Hospital Nnewi over a one year period, January to December 2010. Patients' biodata and medical history were obtained, detailed physical examination done and each patient had a chest X-ray, 12 lead ECG, urinalysis, serum urea and creatinine assay done. Ethical clearance was obtained from the Ethical Review Board of our institution and data analysed using SPSS-version 16. *Results*. There were 86 males and 64 females with mean age 62.7 ± 12.5 years. The mean blood pressures were systolic 152.8 ± 28.5 mmHg and diastolic 94.3 ± 18 mmHg. 84.7% had blood pressure ≥140/90 mmHg on presentation. The mean GFR was 70.1 ± 31.3 mls/min. 76% of subjects had GFR <90 mls/min and no statistical significant difference between males and females, *P* = 0.344. The mean serum urea was 7.2 ± 51 mmol/L while the mean serum creatinine was 194 ± 416.2 mmol/L. *Conclusions*. This study has demonstrated that majority of patients presenting with hypertension associated congestive cardiac failure have some degree of chronic kidney disease.

## 1. Introduction

Congestive heart failure (CHF) is common in North America with the prevalence being approximately 2% in adults older than 45 years [1]. While coronary artery disease (CAD) is a major contributor to the development of congestive cardiac failure in hypertensive patients in Western societies [2], hypertension is the leading cause of cardiac failure in Nigeria [3] and the most common complication of hypertension among patients admitted into wards in South East part of Nigeria [4]. Hypertension is recognized as a leading cause of the global burden of disease and in most African countries, hypertension is the commonest noncommunicable disease [5, 6].

Renal dysfunction is recognised as an independent risk factor for morbidity and mortality in congestive cardiac failure [7, 8]. The association between cardiac failure and chronic kidney disease is well established and referred to as the cardiorenal syndrome by some authors [9]. Obasohan and Ajuyah [10] had earlier reported from the Niger-Delta area of Nigeria that renal dysfunction is independently associated with the development of cardiac failure in hypertensive patients. There are no reports known to the authors on chronic kidney disease in patients with cardiac failure in the South Eastern part of Nigeria. This necessitated carrying out this study in this part of Nigeria to determine renal function in patients with hypertension associated cardiac failure.

## 2. Methodology

This is a cross-sectional study involving 150 adult patients aged 18–80 years in hypertension associated congested cardiac failure. They were recruited consecutively from medical outpatient clinic and the medical wards of Nnamdi Azikiwe University Teaching Hospital Nnewi, Anambra State, South East Nigeria. A diagnosis of hypertension associated congestive cardiac failure was made using the Framingham criteria [11] for diagnosis of congestive cardiac failure and the presence of at least three markers of chronic hypertensive vascular disease and exclusion of other causes of congestive cardiac failure as previously suggested by Araoye and Olowoyeye [12]. This clinical approach to diagnosis of congestive heart failure was adopted because at the time of the study there was no echocardiography machine in the institution at the time of the study. Patients' personal data and medical history were collected using a structured questionnaire administered by the researchers. Detailed physical examination and laboratory investigations were done on each patient. The investigations included a chest X-ray, 12 lead Electrocardiogram, renal imaging (to exclude those with preexisting renal disease), serum urea, creatinine and fasting venous blood glucose, and urine analysis. (Proteinuria was determined by dipstick. Though the method most commonly used to measure urinary protein relies on 24-hour urine collection, which is the gold standard, it is time consuming, cumbersome, and often inaccurate and imprecise and hence a spot urine examination for protein would be more acceptable and less time consuming) [13]. The blood samples were collected prior to the commencement of treatment for heart failure. The patients' glomerular filtration rate (GFR) was calculated using the computer software GFR Calculator (CKD-EPI formula) from the Kidney Health Australia [14] of modification of diet in renal disease (MDRD) which has been validated by the National Kidney Foundation [15]. Renal impairment was defined by GFR <90 mLs/min and was stratified as recommended by the (United States) National Kidney Foundation [15].

Inclusion criteria for the study were

- patient's age between 18 and 80 years;
- congestive cardiac failure using the Framingham criteria [11] and the presence of at least three markers of chronic hypertensive vascular disease;
- congestive cardiac failure using the Framingham criteria [11] and the presence of blood pressure greater than 140/90 mmHg.

Exclusion criteria for the study were

- obvious cause of congestive cardiac failure such as valvular heart disease, cardiomyopathy, and ischemic heart disease;
- congestive cardiac failure with other comorbidities such as diabetes mellitus, human immunodeficiency virus infection (HIV), chronic obstructive airway disease, infections of the urinary tract, trauma, or taking nonsteroidal anti-inflammatory agents (NSAID), or being involved in strenuous exercise prior to urine production;
- those found on renal imaging to have preexisting renal disease or suffering from diseases like multiple myeloma, systemic lupus erythematosus (SLE), and so forth.

Ethical clearance for the study was obtained from the Ethical Review Board of the Nnamdi Azikiwe University Teaching Hospital Nnewi, Anambra State, South East Nigeria. 

 Statistical analysis was carried out using SPSS version 16. Student's *t*-test was used for continuous variables, and *χ* ^2^ test was used for categorical variables. In the analyses a *P* value of <0.05 was considered statistically significant.

## 3. Results

There were 86 (57.3%) males and 64 (42.7%) females. The mean age of the subjects was 62.7 ± 12.6. The majority (56.7%) were traders. The educational attainment of the subjects is shown in Table 1.

127 (84.7%) of the patients were aware that they had hypertension. The mean systolic blood pressure was 152.8 ± 28.5 mmHg while the mean diastolic blood pressure was 94.3 ± 18 mmHg. The mean duration of hypertension was 7.6 ± 8.9 years. 37 (27.3%) of the subjects had blood pressure less than 140/90 mmHg on presentation.

The mean GFR of the study population was 70.1 ± 31.3 mLs/min. 114 (76%) of the subjects had renal impairment (GFR < 90 mLs/min). Mean serum urea concentration was 7.2 ± 5.1 mmol/L while mean serum creatinine was 194 ± 416.2 mmol/L.  Table 2 shows a comparison of the renal indices between the male and female subjects. There was no statistically significant difference between the mean GFR, serum urea, creatinine of the male and female subjects (*P* = 0.344, 0.159, and 0.100, resp.). 

Proteinuria was found in 52 (34.6%) of the subjects.  Table 3 shows the educational attainment.  Table 4 shows the degree of proteinuria found in the study population.

## 4. Discussion

Our study found impaired renal function in 76% of the subjects which confirms the assertion that chronic kidney disease is common in patients with congestive cardiac failure. The majority of subjects (43.3%) had mild chronic kidney disease (GFR equal or greater than 60 mls/min and GFR less than 90 mls/min). This compares favorably with the findings of 72% prevalence in Blacks and 69% in Whites found by Smith et al. [16]. The 76% prevalence of chronic kidney disease found in our study is higher than that of Smith et al. [17] who reported chronic kidney disease of 63% found in patients in a meta-analysis of 16 studies involving 80,098 patients in congestive cardiac failure. The meta-analysis involved studies with different definitions of chronic kidney disease as some of the studies used GFR <90 mLs/min, others used GFR <53 mLs/min and one study used cystatin C level above 1.03 mg/dL. This may be responsible for the lower prevalence in the meta-analysis. In another study Go et al. [18] reported that about 50% of patients with chronic heart failure had impaired renal function based on estimated GFR <60 mLs/min.

 Obasohan and Ajuyah [10] in the Niger-Delta area of Nigeria found renal impairment in 55% of patients in hypertensive heart failure. This lower prevalence might be attributed to the use of creatinine levels above 1.5 mg/dL to define renal dysfunction instead of a cut-off point for GFR as used in this study. The study by Obasohan and Ajuyah has a significantly smaller sample size (55 patients) compared to our study with a sample size of 150 patients.

The high values may also reflect the relatively lesser contribution of coronary artery disease to heart failure in Black hypertensive patients than Caucasian patients [19].

 Chronic kidney disease in congestive cardiac failure patients has become increasingly recognized as an independent risk factor for morbidity and mortality [20]. Hypertension induced renal dysfunction is quite common in the Nigerian population as shown by Nwankwo et al. [21]. In their study they showed in a population of hospitalized hypertensive patients a prevalence of chronic kidney disease of 45.5%. The high prevalence of chronic kidney disease in our hypertensive patients when compared with patients from UK and USA could be explained in a number of ways. (a) The high prevalence may be as a result of the study population being entirely hospitalized hypertensive patients who presented for care as a result of end organ damage from hypertension. (b) As has been shown [22, 23] the hypertensive African American has substantially a higher risk of chronic kidney disease than his Caucasian American counterpart. It is becoming increasingly apparent that gene based differences in disease profile may contribute to the disproportionate burden of CKD across populations and the genetic relatedness of our population to the African American may contribute to the high rate of impaired renal function [24, 25]. (c) Socioeconomic factors such as poverty and low income with the consequent limited access to health care and poor urban housing have been reported to contribute to the incidence and prevalence of hypertension induced chronic kidney disease. (d) Poor control of hypertension as only 27.3% of our subjects had blood pressure >140/90 mmHg on presentation. This level of poor control could explain the high prevalence of chronic kidney disease [26] in those hypertensives that had developed congestive cardiac failure as poor control of hypertension is associated with the development of hypertensive complications of which renal impairment is one [27, 28].The mechanism underlying the occurrence of kidney dysfunction in cardiac failure is still not completely understood and is said to be complex and multifactorial as both the heart and the kidney work together to regulate blood pressure, vascular tone, diuresis, natriuresis intravascular volume homeostasis, peripheral tissue perfusion, and oxygenation [29]. It is well known that decline in cardiac function causes decrease in tissue perfusion, and thus, adversely affects renal perfusion. In the setting of congestive cardiac failure the most common type of renal dysfunction is prerenal azotaemia [30]. The reduction in renal perfusion and the consequent fall in GFR lead to reflex activation of the renin-angiotensin-aldosterone system (RAAS) resulting in tubular retention of salt and water [31]. In addition, some of the neurohormonal and inflammatory activation commonly observed in HF patients probably also contributes to renal dysfunction. The chronic inflammatory state that is present in both chronic kidney disease and congestive cardiac failure, in turn, can cause reactive oxygen species (ROS) production by activating leukocytes to release their oxidative contents [32]. Increased ROS production and lower availability of nitric oxide (NO), resulting in NO/ROS imbalance, may increase activity of preganglionic sympathetic neurons and stimulate RAAS directly by damaging the renal tubular or interstitial cells or by afferent vasoconstriction with chronic inhibition of NO synthesis [33]. Data also suggest that the high renal venous pressure contributes to vasomotor nephropathy and further amplifies chronic kidney disease in cardiac failure [34]. 

## 5. Recommendation

In view of the high prevalence of chronic kidney disease in hypertension associated congestive cardiac failure it is recommended that health awareness campaigns targeted at vulnerable groups be initiated, for the detection of chronic kidney disease in those with congestive cardiac failure and hypertension using the media (radio and television), health talks at town/village meetings, churches, and so forth.

Also clinicians who provide care should be encouraged to screen hypertensive patients and those in congestive cardiac failure periodically by estimating the GFR to detect a decline in renal function and measures instituted to reduce the morbidity and mortality associated with chronic kidney disease in congestive cardiac failure.

## 6. Conclusions

This study showed that there is a high prevalence of chronic kidney disease in patients in congestive cardiac failure and is in agreement with similar studies done in other countries. There are differences in the prevalence of chronic kidney disease in congestive cardiac failure in different studies done due to nonuniformity in the criteria for defining chronic kidney disease. 

## Figures and Tables

**Table 1:** Educational attainment of respondents.

	No. of patients (*N* = 150)	Percent
No. formal education	47	31.3
Primary school	68	45.3
Secondary school	17	11.3
Tertiary school	18	12.0

**Table 2:** Comparison of renal function indices of males and females.

Variable	Sex	No.	Mean	Standard deviation	*t*-test	*P* value
Serum urea	Male	86	7.73	6.06	1.41	0.159
	Female	64	6.52	3.64		
Serum creatinine	Male	86	243.78	525.25	1.67	0.100
	Female	64	130.58	173.27		
GFR	Male	86	72.22	35.17	0.948	0.344
	Female	64	67.32	25.16		

**Table 3:** Showing the GFR ranges in the subjects.

GFR (*N* = 150)	No.	Percentage
≥90	36	24
60–89	65	43.3
30–59	34	22.7
15–29	6	4
<15	9	6

**Table 4:** Pattern of proteinuria in the subjects.

*N* = 150	Frequency	Percentage (%)
Nil (<10 mg/dL)	98	65.3
Trace		
(>10 mg/dL, <30 mg/dL)	9	6
(>30 mg/dL, <100 mg/dL)	25	16.7
(>100 mg/dL, <500 mg/dL)	11	7.3
(>500 mg/dL)	7	4.7
